# The role of glucose and insulin in the metabolic regulation of growth hormone secretion

**DOI:** 10.14341/probl12660

**Published:** 2021-01-21

**Authors:** E. L. Sorkina, V. V. Chichkova, I. A. Sklyanik, M. V. Shestakova, G. A. Mel'nichenko, A. Barkan

**Affiliations:** Endocrinology Research Centre; Endocrinology Research Centre; Endocrinology Research Centre; Endocrinology Research Centre; Endocrinology Research Centre; University of Michigan, Ann Arbor

**Keywords:** growth hormone, insulin, glucose, oral glucose tolerance test

## Abstract

The exact physiological basis for the suppression of growth hormone secretion by oral glucose intake remains unknown, despite the widespread use of the oral glucose tolerance test in endocrinology. Lack of growth hormone suppression by glucose occurs in about a third of patients with acromegaly, as well as in other disorders. It is currently known that the secretion of growth hormone is affected by various factors, such as age, gender, body mass index, and the redistribution of adipose tissue. There is also evidence of the impact of overeating as well as being overweight on the secretion of growth hormone. It is known that both of these conditions are associated with hyperinsulinemia, which determines the possibility of its predominant role in suppressing the secretion of growth hormone. The purpose of this review is to discuss the accumulated data on the isolated effects of hyperglycemia and hyperinsulinemia on growth hormone secretion, as well as other metabolic regulators and conditions affecting its signaling. Understanding of the pathophysiological basis of these mechanisms is essential for further research of the role of glucose and insulin in the metabolic regulation of growth hormone secretion. However, the studies in animal models are complicated by interspecific differences in the response of growth hormone to glucose loading, and the only possible available model in healthy people may be the hyperinsulinemic euglycemic clamp.

## Abstract (ru)

Точные физиологические основы подавления секреции гормона роста пероральным приемом глюкозы на сегодняшний день остаются неизвестными, несмотря на широкое применение перорального глюкозотолерантного ­теста в эндокринологии. Отсутствие подавления гормона роста глюкозой встречается примерно у трети пациентов с акромегалией, а также при других расстройствах. В настоящее время известно, что на секрецию гормона роста воздействуют различные факторы, такие как возраст, пол, индекс массы тела и перераспределение жировой ткани. Также появляются данные о влиянии на секрецию гормона роста не только избыточной массы тела, но и переедания. Как ­известно, оба этих состояния связаны с гиперинсулинемией, что определяет возможность ее преимущественной роли в подавлении секреции гормона роста. Целью данного обзора является обсуждение накопленных данных об изолированном влиянии на секрецию гормона роста гипергликемии и гиперинсулинемии, а также других метаболических регуляторов и состояний, влияющих на его сигналинг. Понимание патофизиологических основ этих механизмов необходимо для дальнейших исследований роли глюкозы и инсулина в метаболической регуляции секреции гормона роста. При этом важно учитывать, что исследования на животных моделях затруднены межвидовыми различиями в ответе гормона роста на нагрузку глюкозой, и единственной возможной доступной моделью у здоровых людей авторам представляется использование гиперинсулинемического эугликемического клэмп-теста.

## GROWTH HORMONE SECRETION CONTROL UNDER NORMAL CONDITIONS — WHAT IS CURRENTLY KNOWN?

Growth hormone (GH), or somatotrophin, is a single-chain polypeptide consisting of 191 amino acids. GH stimulates the physical growth and development of humans and animals, and also plays an important role in maintaining the proper functioning of the body, such as exerting various metabolic effects, participating in the processes of reproduction and aging [1][2]. The main metabolic effect of GH is the lipolysis stimulatory effect and lipids acidification [3-5].

GH is produced by the anterior lobe of the pituitary gland, by somatotrophs in the pulsatile mode: the peak of its secretion falls in the late evening and early hours (circadian rhythm), simultaneously with the onset of the slow-wave sleep phase, and the amplitude and frequency of secretory GH pulses are influenced by various factors, such as age, reproductive hormones, and nutritional status [6]. The classical GH secretion control is carried out by two hypothalamic hormones: the GH — releasing hormone (GHRH) and somatostatin, that have a stimulating and inhibitory effect on the somatotrophs, respectively (Fig. 1). Pulsatile GHRH releases are caused by the episodes of hypothalamic GHRH secretion. It has been shown that passive immunoneutralization of GHRH in rats, as well as a blockade of GHRH receptors by a specific antagonist in humans, eliminates the generation of the GH pulses [7][8].

**Figure:**
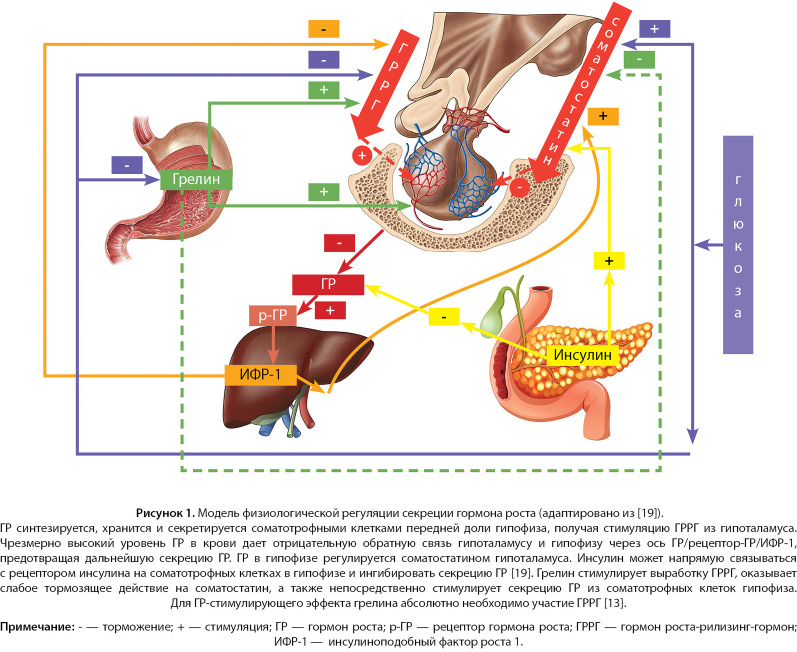
Figure 1. The model of physiological control of growth hormone secretion (adapted from [19]) GH is synthesized, kept, and secreted by the pituitary gland somatotrophs, receiving GHRH stimulation from the hypothalamus. An excessively high level of GH in the blood results in down regulation to the hypothalamus and pituitary gland via the GH/GH-receptor/IGF-1 axis, preventing further GH secretion. GH in the pituitary gland is regulated by the somatostatin of the hypothalamus. Insulin can directly bind to the insulin receptor on pituitary gland somatotrophs and inhibit the GH secretion [19]. Ghrelin stimulates production of GHRH, has a weak inhibitory action on somatostatin, and also directly stimulates the GH secretion from the pituitary gland somatotrophs. For the GH-stimulating effect of ghrelin, the participation of GHRH is absolutely critical [13].Note: — inhibition; + — stimulation; GH — growth hormone; GH-r — growth hormone receptor; GHRH — growth hormone-releasing hormone; IGF-1 — insulin-like growth factor 1.

Basal GH secretion is determined by the somatotrophs mass and the somatostatin tone. Somatostatin also reduces the pituitary response of GH to GHRH [9], and basal GH secretion [10], and probably controls the circadian rhythm of GH secretion [11]. All the known GH stimulation tests (insulin hypoglycemia, clonidine, arginine, pyridostigmine, L-DOPA, GH -releasing peptide-6) require the presence of endogenous GHRH for their effect [12][13]. One more known activator of the GH secretion is ghrelin, an intestinal peptide and ligand of the GH secretagogue receptor. Ghrelin also directly contributes to the production of GH by somatotrophs, in addition to its stimulating effect on the secretion of GHRH and, less prominently — somatostatin [14]. Insulinlike growth factor 1 (IGF-1) has a significant inhibitory effect on the secretion of GH by the mechanism of down regulation within the pituitary gland and hypothalamus. IGF-1 can inhibit both spontaneous and GHRH-stimulated GH release and have a stimulating effect on somatostatin neurons [15]. GH secretion is also influenced by a number of factors, such as neuropeptides, neurotransmitters, peripheral hormones such as thyroxine, glucocorticoids, sex steroids, leptin, and various metabolic signals — all of which in combination form a complex control network of GH secretion [16-18]. One of the key factors influencing the secretion and signaling of GH is glucose and insulin [19][20].

## FUNCTIONS OF THE GH SECRETORY PROFILE

GH performs two essential functions: stimulating growth by increasing the synthesis of IGF-1 in all tissues, and controlling metabolism (primarily — lipolysis). With sufficient nutrition and in the presence of adequate insulin secretion, the metabolic function of GH is not expressed, but with fasting (suppressed insulin secretion) GH becomes the essential metabolic control, supplying lipolysis products as energy substrates that support vital functions [21].

The induction of tissue IGF-1 and, as a result, somatic growth also depend on the manner of presentation of GH (pulse or continuous basal) to peripheral tissues. Experiments in rats and humans revealed diametrically opposite responses. Only GH pulse administration increased the levels of tissue ribonucleic acid (mRNA) of the insulin-like growth factor-1 [22] in rats, while this effect in humans was expressed only with the GH continuous infusion, and lipolysis was stimulated by the GH pulse administration, copying the pulse aspect of the GH endogenous secretion [22][23].

## THE EFFECT OF GLUCOSE ADMINISTRATION, ACUTE AND CHRONIC HYPERGLYCEMIA ON THE SECRETION AND GROWTH HORMONE SIGNALING

The effect of glucose on GH secretion was shown in the early 1960s and has since been confirmed by various authors. Thus, it is well known that hypoglycemia has a stimulating effect on the GH secretion, and therefore the test with insulin hypoglycemia is used in clinical practice to assess the adequacy of GH secretion, in particular, for the diagnosis of GH insufficiency. Simultaneously, oral glucose administration suppresses the GH secretion and allows us to evaluate the inhibitory control of the GH secretion [24]. However, some aspects of the pathogenetic foundation of the effect of glucose on the secretion of GH have yet to be studied.

The study of this problem in animal models is complicated by interspecies differences in the response of GH to glucose loading and to fasting. In humans a sharp drop in blood glucose levels stimulates the GH secretion, but in rat pituitary it neither changes nor lowers. [25]. With prolonged exposure to high glucose concentrations on cultured rat cells of the anterior pituitary, it was concluded that the glucose level in the surrounding solution directly modulates the GH release, as well as reducing its response to somatostatin [26].

During the subsequent studies in rat models in vivo, it was determined that both acute hypo-and hyperglycemia stimulate somatostatin mRNA, whereas GHRH mRNA is stimulated only by hyperglycemia [27].

Chronic hyperglycemia, such as diabetes mellitus (DM), also has the opposite effect on the production of GH in humans and in rats.

In type 2 diabetes mellitus (T2DM), the data is contradictory. Spontaneous and GHRH-stimulated GH secretion in rats may increase, remain stable, or decrease [28]. One of the main determinants of the differences in this response is obesity. Patients with T2DM and obesity show a significantly lower GH response to GHRH in comparison to patients with T2DM without obesity [29][30].

Type 1 DM is accompanied by increased GH pulse secretion and increased GH release after the GHRH administration [31][32]. In rodents, DM probably reduces the GH pulsatile secretion and weakens its secretory response to GHRH. Hyperglycemia appears to directly affect the pituitary somatotrophs, reducing the GH release in response to GHRH or increasing the release of somatostatin [33–35]. The lowered GH response is restored after treatment with somatostatin antiserum or pentobarbital anesthesia, which presumably suppresses somatostatin release [36]. In streptozotocin-induced DM, GH secretion in rats decreases along with a decrease in GHRH mRNA and somatostatin mRNA [37]. However, it is important to note that a certain decrease in the release of hormones by the pituitary gland when high doses of streptozotocin are administered may be secondary to the toxic destruction of somatotrophs [38].To summarize the above, it may be concluded that the automatic use of data obtained in rats (the most common model) to interpret the dynamics of GH in humans may not be appropriate.

## THE EFFECT OF OTHER METABOLIC REGULATORS AND CONDITIONS ON GROWTH HORMONE SECRETION

One of the GH secretion controls is amino acids, in particular, arginine. In vivo and in vitro investigations have shown that bolus administration of arginine increases the expression of the GH gene and induces insulin resistance [39].

Increased levels of free fatty acids (FFA) in obese patients also play a role in the pathogenesis of the GH hyposecretion in obesity. To clarify their role, Maccario M. et al. have studied the effect of a decrease in the level of FFA plasma induced by oral administration of an anti-lipolytic medicine (acipimox) on the response of GH to GHRH individually or in combination with arginine. The data obtained indicated that an acute decrease in the level of FFA in the blood plasma in obese patients restores their somatotropic reactivity, while it does not affect the GH secretion in healthy individuals [40]. The paradoxical reactions of the GH to increased FFA are insufficiently studied: their pharmacological increase blocks the GH secretion [41], while during fasting their endogenous increase is accompanied by stimulation of the GH secretion [42].

Many studies have demonstrated down regulation of GH levels in humans with high body mass index [43], especially in obese patients (with a body mass index of more than 30 kg/m2) [44]. In general, investigations of GH secretion in obesity have demonstrated the decrease in both spontaneous [45] and stimulated GH secretion [46]. However, according to the data obtained later by Anderwald C. H. et al., it is insulin resistance, and not the body mass index, that has a significant effect on GH levels — both in the fasting and in the oral glucose tolerance test (OGTT) [47].

It is important to note that in patients with impaired GH secretion, both in acromegaly and in GH deficiency, insulin sensitivity is impaired, and this is not related to fat accumulations in any way. In acromegaly, there is a decrease in the amount of fat mass and an increase in insulin resistance, and mice with the isolated GH deficiency are characterized by an increased sensitivity to insulin, despite the excess fat mass. In people with GH deficiency, an increase in the amount of body fat accumulation and a decrease in the mass of free fatty acids is determined, but the results regarding insulin sensitivity are contradictory, since it is assumed that there are other factors that affect insulin resistance [48].

There is data on the suppression of GH secretion not only in obesity, but also in relation to overeating [49]. As is commonly known, both of these conditions are associated with hyperinsulinemia, which demonstrates the possibility of its predominant role in suppressing the GH secretion. During the experiments with overeating, it was found that overconsumption for 2 days, even before the appearance of any weight gain, suppresses GH secretion, which in the early stages counteracts insulin resistance and hyperlipidemia, and in turn reduces the risk factors for cardiovascular diseases [49].

## GH AND IGF-1: DOWNREGULATION

With the purpose of confirming the direct relationship between the GH secretion and IGF-1, Hartman M. L. et al. in their works conducted a study of the administration of recombinant human IGF-1 (rIGF-1) to men in a state of euglycemia but fasting for 32 hours, which normally increases GH secretion. As a result of human rIGF-1 infusion, the rate of GH secretion decreased after ½ min and remained suppressed after that. During the infusion of saline (in the control group), the rate of GH secretion remained increased. It was concluded that the GH secretion, increased in the fasting state, and is rapidly suppressed in the state of euglycemia with the help of low-dose infusion of human rIGF-1. This effect of rIGF-1 is probably mediated through IGF-1 receptors, regardless of its insulin-like metabolic action [50]. Further studies relating to fasting and the rIGF-1 administrating, Chapman I.M., Hartman M.L. et al. determined the recovery time of GH secretion after its suppression. The results show that the blood GH was maximally suppressed within 2 hours and remained suppressed for 2 hours after infusion of human rIGF-1. The close temporal relations between suppression of GH and a drop in free IGF-1 concentration, as well as the absence of any relationship with total IGF-1 concentrations, suggested that unbound (free) IGF-1 is the main component of IGF-1 responsible for this suppression [51]. It is important to note that the age of the subjects also affects the suppression of GH after the rIGF-1 administration. Chapman I.M. et al. concluded that the ability of exogenous rIGF-1 to suppress GH concentration in the blood serum reduces with increasing age. This suggests that increased sensitivity to endogenous down regulation of IGF-1 is not the reason for the decrease in GH secretion that occurs with aging [52].

## THE INFLUENCE OF INSULIN ON GROWTH HORMONE SECRETION AND SIGNALING

The molecular mechanisms of insulin suppression of GH secretion are currently not fully defined. The effect of insulin physiological doses on the levels of triiodothyronine (T3)- stimulated GH mRNA in rat pituitary tumor cells had been studied earlier. Insulin (7 nmol/ L) selectively suppressed T3- stimulated GH mRNA levels in pituitary tumor cells by 58%. This suppressive effect of insulin occurred independently of protein synthesis and appeared to be mediated at both the transcriptional and posttranscriptional levels [53][54].

It is known that control over gene transcription is usually provided by transactive transcription factors that bind to superior regulatory elements. Since insulin regulates the transcription of the GH gene, some studies have demonstrated the binding of insulin through an insulin-induced DNA-binding protein to the human GH gene, which suggests a transactive role for insulin in mediating the expression of the polypeptide hormone gene [55]. Studies by Prager et al. on the effects of insulin on the expression of the transfected human GH gene were carried out. A fragment of the human GH gene was propagated in pUC18 and transfected with calcium phosphate shocking into HeLa and GC cells, respectively. Transfected cells, grown in serum-free medium for 72 hours, expressed human GH. The results show that insulin (0.7-7 nM) suppressed both basal and hydrocortisone-stimulated (100 nM) expression of newly synthesized 22-kDa GH in a dose-dependent manner. Insulin (7 nM) also suppressed basal and hydrocortisone-stimulated GH mRNA transcripts in transfected cells. The GH promoter determined insulin sensitivity through the chloramphenicol acetyltransferase reporter gene. Therefore, cis-acting control sequences located in the 5’-flanking region of the 497 base pair of the human GH gene appear to be critical for the response of the human GH gene to the insulin signaling [56]. In addition, in recent works, the role of insulin synthesized in the paraventricular hypothalamic nucleus in the control of GH secretion by the pituitary gland has been discussed. As can be seen from the above, the pathogenetic relationship between GH secretion and insulin is not in doubt, but the details of this relationship require further study [57].

## MECHANISMS OF THE ORAL GLUCOSE TOLERANCE TEST EFFECT ON THE SUPPRESSION OF GH SECRETION: THE ROLE OF HYPERINSULINEMIA AND HYPERGLYCEMIA

Despite the fact that the effect of oral glucose administration on GH secretion was discovered more than 50 years ago [24], the exact mechanism of this effect remains undefined. Standardized 75 g glucose OGTT is a widely used method for diagnosing carbohydrate metabolism disorders such as impaired glucose tolerance and DM, but it is also used to confirm or exclude the diagnosis when clinically suspected acromegaly [58]. Suppression of GH in OGTT below 1 ng/ml within 2 h after loading is currently considered a criterion for excluding acromegaly, in addition to IGF-1 levels within the age-related reference range [59].

It is assumed that the suppression of GH in OGTT is associated with a glucose-dependent increase in the level of somatostatin.

This assumption is based on the data that in healthy people the GH secretion in response to GHRH or GH secretagogue decreases after oral glucose administration [60][61]. In addition, the acetylcholinesterase inhibitor pyridostimine neutralizes the suppression of GH by glucose, purportedly by suppressing the secretion of somatostatin by the hypothalamus [62].

It was later suggested that ghrelin plays a role in regulating the effect of glucose on GH [63]. According to the results of multivariant analysis in a study by Pena-Bello L. et al., ghrelin was the only predictor of fasting and peak GH levels during oral glucose loading in women [64]. Interestingly, some authors demonstrated that there is no relationship between the maximum suppression of GH specifically by glucose administration, revealing a similar decrease in GH levels after drinking water and even after random measurements, demonstrating that glucose rather inhibits spontaneous GH releases [65][66].

Despite the insufficient study of the pathophysiological foundation of this process, it has recently been suggested that the interaction between somatostatin and GHRH is impaired, and an association has been shown between ectopic pituitary expression of the glucose-dependent insulinotropic polypeptide receptor [67]. The least pronounced suppression of GH in OGTT is observed in adolescence [68]. The levels of GH secretion decrease with age — by 14% every 10 years, starting from the age of 20 [45], and it has also been shown that the levels of maximally suppressed GH in OGTT also decrease with age [69]. This is most likely due to the relative deficiency of GHRH and ghrelin and increased secretion of somatostatin in the elderly [70]. For women, compared with men, a less pronounced suppression of GH in OGTT is characteristic both in a healthy population [71] and among patients with acromegaly [72]. This is assumed to be associated with an initially higher basal GH level in women, as has been shown in some investigations [73][74]. In patients with acquired lipodystrophy due to highly active anti-retroviral therapy for HIV infection, there was no reverse increase in GH levels during 2-hour OGTT, which suggests the effect of redistribution of subcutaneous adipose tissue on a longer suppression of GH [75].

However, an obvious question in relation to the OGTT mechanism of action that has never been asked is which component of OGTT is responsible for the suppression of GH – insulin or glucose? In healthy individuals, oral glucose administration is accompanied by an increase in glucose and insulin levels and a suppression of the GH levels in the blood for 2–3 hours, and then a delayed increase in GH levels is observed 3–5 hours after glucose administration [76]. So the differentiation between the specific inhibitory effects of insulin and glucose on GH becomes almost impossible to determine. Conducting OGTT in type 1 diabetes patients with lack of insulin secretion may be more informative, but the ethical limitations of such an experiment are obvious. The hyperinsulinemic normoglycemic clamp may be the only viable model.

The fundamental mechanisms that control the GH secretion and its actions are still poorly defined and controversial. The GH secretion control mechanisms by pharmacological interventions have been conducted in numerous investigations with dopaminergic, adrenergic, and cholinergic drugs [77][78]. Since all of them have neural hypothalamic points of application, their investigations allowed us to infer neuroregulatory mechanisms of the GHRH secretion and, to a lesser extent, of somatostatin [79]. The use of an antagonist to the GHRH receptor [7] and other physiological protocols [12] have already demonstrated the effect of these medicines on the GHRH and somatostatin secretion. Their relevance to the physiological, endogenous control of GH secretion remains in doubt: in everyday life, we do not regulate the GH release or suppression by administering dopaminergic or antidopaminergic drugs, β-blockers, or the anticholinergic pyridostigmine. Paradoxically, the GH control by metabolic factors that are constantly present in our body has never been studied with sufficient scientific accuracy. How does oral glucose administration suppress GH — by increasing glucose or by increasing insulin — is one question that remains unanswered. We assume that this issue can be resolved using clamp technology, which has never been applied. This is extremely relevant in understanding the role of GH in the DM pathogenesis.

## CONCLUSION

An analysis of the literature used led to the conclusion that, despite the fact that the effect of glucose on GH secretion has been known since the middle of the last century, the specific mechanisms of the GH secretion suppression by oral glucose administration remain undefined. The pathogenetic relationship between the GH secretion and insulin secretion both during glucose loading and in other conditions represented of insulin resistance is not in doubt, but requires further study. Taking into consideration the interspecies differences in the GH response to glucose load, it is possible to evaluate the isolated effect of hyperinsulinemia on the regulation of GH secretion only during the hyperinsulinemic euglycemic clamp test. The study of the causal relationship and the various factors effect on the outcome during OGTT will help to better understand the mechanism of GH secretion, which, in turn, will make it possible to more accurately interpret the results, including the patients with a “paradoxical response”.
